# Symptoms of COVID-19 in children

**DOI:** 10.1590/1414-431X2022e12038

**Published:** 2022-06-13

**Authors:** M.M. Melo, M.M.R. Neta, A.R.S. Neto, A.R.B. Carvalho, R.L.B. Magalhães, A.R.M.C. Valle, J.H.L. Ferreira, K.M.J. Aliaga, M.E.B. Moura, D.R.J. Freitas

**Affiliations:** 1 Programa de Pós-Graduação em Enfermagem, Departamento de Enfermagem, Universidade Federal do Piauí, Campus Universitário Ministro Petrônio Portella, Teresina, PI, Brasil; 2 Departamento de Enfermagem, Universidade Federal do Piauí, Campus Universitário Ministro Petrônio Portella, Teresina, PI, Brasil; 3 Programa de Pós-Graduação em Ciências e Saúde, Universidade Federal do Piauí, Campus Universitário Ministro Petrônio Portella, Teresina, PI, Brasil

**Keywords:** COVID-19, SARS-CoV-2, Symptoms, Children, Diagnosed cases

## Abstract

The aim of this study was to review the symptomatic manifestations of COVID-19 in children in the scientific literature. An integrative review of studies published between December 2019 and September 5, 2021, from the Medical Literature Analysis and Retrieval System Online, Web of Science, Scopus, Literatura Latino-Americana em Ciência de Saúde, and Base de Dados de Enfermagem databases, was carried out to answer the following research question: What symptomatic manifestations does COVID-19 cause in children?”. Twenty articles were included. The main symptoms described were fever, cough, diarrhea, vomiting, sore throat, dyspnea, headache, abdominal pain, malaise, and weakness or tiredness. The findings of this review can contribute to the diagnosis and clinical decision-making of the health team by providing information that facilitates the identification of COVID-19 in the target population, favoring early identification, better care, and consequently a better prognosis.

## Introduction

In December 2019, the Municipal Health Commission in Wuhan city, Province of Hubei, China, identified a new disease caused by a coronavirus (COVID-19). On January 13, 2020, health authorities reported the first case of COVID-19 outside China in Thailand. Because of the alarming spread, severity of the disease, and large number of people affected, the World Health Organization (WHO) declared COVID-19 a pandemic on March 13, 2020 (1). As of January 28, 2022, approximately 364,191,494 people were affected, with 5,631,457 deaths worldwide (2). In addition, according to data from the United Nations Children's Fund (UNICEF) published in January 2022, there were about 12,300 deaths in children and teenagers under 20 years of age, 42% of which were in children aged 0 to 9 years (3).

The novel coronavirus was named SARS-CoV-2, an acronym for Severe Acute Respiratory Syndrome Coronavirus-2 (4). It has been described that the symptoms caused by the disease can affect different systems in adults (5). Although children get sick less often than adults, they can transmit the virus even if they are asymptomatic or have mild disease symptoms. However, some children can become seriously ill and require hospitalization, intensive care, or mechanical ventilation, and in rare cases, they may even die (6).

Thus, in April 2020, the British Pediatric Association issued an alert to government health agencies reporting the identification of a new clinical presentation in children possibly associated with COVID-19, called Multisystem Inflammatory Syndrome in children (MIS-C). This syndrome can lower blood pressure and cause fluid to build up in the lungs and other organs, making intensive care necessary to support primarily heart and lung functioning. Therefore, it became evident that although children are not as severely affected as adults and the elderly, they still deserve attention for proper clinical management, seeking a better prognosis during and after infection (7).

As this is a new disease, evidence-based discussions are needed to address aspects that are still unknown or little discussed, such as the relationship between children and SARS-CoV-2. Thus, this integrative review aims to review the symptomatic manifestations of COVID-19 in children in the scientific literature.

## Material and Methods

This integrative literature review was conducted in six stages, namely: elaboration of the research question, search and sampling of the literature, definition of the information to be extracted from the selected articles, critical evaluation of the included evidence, interpretation of results, synthesis of knowledge, and presentation of the review (8).

The research guiding question was elaborated using the acronym PVO (P: population; V: variable of interest; O: outcome), defining the following structure: P: Children; V: Symptomatic manifestations; and O: COVID-19 (9). Thus, this investigation was based on the following question: “What symptomatic manifestations does COVID-19 cause in children?”.

The bibliographic survey was carried out between September and October 2021 by two independent reviewers (M.M.M. and M.M.R.N.), and discrepancies were resolved by a third reviewer (A.R.S.N.), through consultation of the Medical Literature Analysis and Retrieval System Online (MEDLINE^®^ via PubMed^®^) databases, Web of Science TM (WOS), Scopus (Elsevier), Literatura Latino-Americana em Ciência de Saúde (LILACS), and Base de Dados de Enfermagem (BDENF) via Biblioteca Virtual em Saúde (BVS) databases. For the operationalization of the search, controlled and uncontrolled descriptors extracted from the vocabularies were selected: Descriptors in Health Sciences (DeCS) and Medical Subject Headings (MESH).

The advanced search form was used to systematize the identification of studies, considering the peculiarities and different characteristics of each database. Controlled and different uncontrolled descriptors were combined using the Boolean descriptor “OR”. For acronyms and synonyms, “AND” was used. The search strategy in the different databases is described in Table 1.

**Table 1:** Search strategies used in the databases.

Database	Search Strategy
MEDLINE (PubMed)	((((“Child”[Mesh]) OR (“child”[All Fields])) OR (“children”[All Fields])) AND (((“Signs and Symptoms”[Mesh]) OR (“signs and symptoms”[All Fields])) OR (“symptoms and signs”[All Fields]))) AND (((((((((((((“COVID-19”[Mesh]) OR (“COVID-19”)) OR (“SARS-CoV-2”[Mesh])) OR (“SARS-CoV-2”)) OR (“covid 19”[All Fields])) OR (“COVID-19 Virus Disease”)) OR (“COVID-19 Virus Infection”)) OR (“2019-nCoV Infection”)) OR (“Coronavirus Disease-19”)) OR (“2019 novel coronavirus disease”[All Fields])) OR (“2019 novel coronavirus infection”[All Fields])) OR (“2019-nCoV Disease”)) OR (“SARS-CoV-2 Infection”))
Scopus (Elsevier)	((TITLE-ABS-KEY (“Child”)) OR (TITLE-ABS-KEY (“Children”))) AND ((TITLE-ABS-KEY (“Signs and Symptoms”)) OR (TITLE-ABS-KEY (“Symptoms and Signs”))) AND ((TITLE-ABS-KEY (“COVID-19”)) OR (TITLE-ABS-KEY (“SARS-CoV-2”)) OR (TITLE-ABS-KEY (“COVID 19”)) OR (TITLE-ABS-KEY (“COVID-19 Virus Disease”)) OR (TITLE-ABS-KEY (“COVID-19 Virus Infection”)) OR (TITLE-ABS-KEY (“2019-nCoV Infection”)) OR (TITLE-ABS-KEY (“Coronavirus Disease-19”)) OR (TITLE-ABS-KEY (“2019 Novel Coronavirus Disease”)) OR (TITLE-ABS-KEY (“2019 Novel Coronavirus Infection”)) OR (TITLE-ABS-KEY (“2019-nCoV Disease”)) OR (TITLE-ABS-KEY (“SARS-CoV-2 Infection”)))
Web of Science	TS=(“Child”) OR TS=(“Children”) AND TS=(“Signs and Symptoms”) OR TS=(“Symptoms and Signs”) AND TS=(“COVID-19”) OR TS=(“SARS-CoV-2”) OR TS=(“COVID 19”) OR TS=(“COVID-19 Virus Disease”) OR TS=(“COVID-19 Virus Infection”) OR TS=(“2019-nCoV Infection”) OR TS=(“Coronavirus Disease-19”) OR TS=(“2019 Novel Coronavirus Disease”) OR TS=(“2019 Novel Coronavirus Infection”) OR TS=(“2019-nCoV Disease”) OR TS=(“SARS-CoV-2 Infection”)
LILACS and BDENF (BVS)	((mh:(“Criança”)) OR (“Criança”) OR (mh:(“Child”)) OR (“Child”) OR (mh:(“Nião”)) OR (“Nião”) OR (“Crianças”) OR (“Niãos”)) AND ((mh:(“Sinais e Sintomas”)) OR (“Sinais e Sintomas”) OR (mh:(“Signs and Symptoms”)) OR (“Signs and Symptoms”) OR (mh:(“Signos y Síntomas”)) OR (“Signos y Síntomas”) OR (“Manifestações Clínicas”) OR (“Observação Clínica”) OR (“Sinais Clínicos”) OR (“Sintoma”) OR (“Sintoma Clínico”) OR (“Manifestaciones Clínicas”) OR (“Observación Clínica”) OR (“Quejas y Síntomas”) OR (“Signo Clínico”) OR (“Síntoma Clínico”) OR (“Síntomas y Quejas”)) AND ((mh:(“Infecções por Coronavirus”)) OR (“Infecções por Coronavirus”) OR (mh:(“Coronavirus Infections”)) OR (“Coronavirus Infections”) OR (mh:(“Infecciones por Coronavirus”)) OR (“Infecciones por Coronavirus”) OR (“COVID-19”) OR (“Doença pelo Novo Coronavírus (2019-nCoV)”) OR (“Doença por Coronavírus 2019-nCoV”) OR (“Febre de Pneumonia por Coronavírus de Wuhan”) OR (“Infecção pelo Coronavírus 2019-nCoV”) OR (“Pneumonia por Novo Coronavírus de 2019-2020”))

The studies found were exported to the EndNote^®^ reference manager software to identify duplicates and gather all publications.

Primary studies on COVID-19 symptoms in children (up to 12 years old) or studies that considered pediatric patients (mean or median age of 12 years), published between December 2019 and September 5, 2021, without language restriction, and diagnosed primarily by reverse transcription-polymerase chain reaction (RT-PCR positive for SARS-CoV-2) were included. Articles with methodological inconsistencies, such as studies without COVID-19 confirmation, reviews, opinion articles, reflection articles, editorials, and articles outside the scope of the research were excluded.

**Figure 1:**
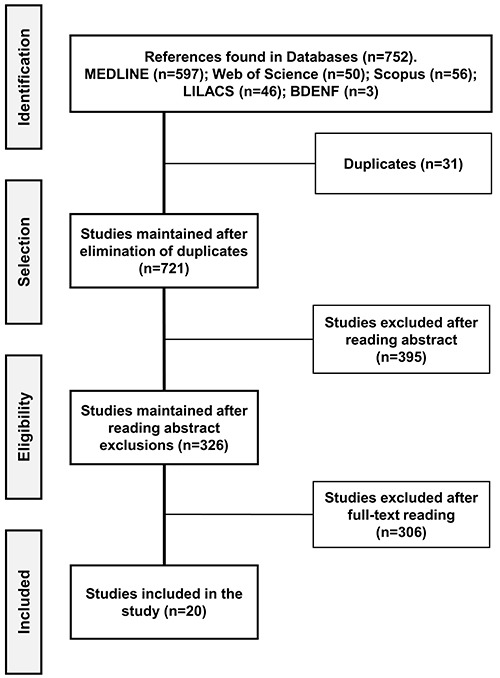
Flowchart of article selection process in the databases.

The selection of studies followed the recommendations of the Preferred Reporting Items for Systematic reviews and Meta-Analyses - extension for Scoping Reviews (PRISMA-ScR) (10), as shown in Figure 1.

The following data were extracted for the identification and characterization of the studies: authors, year of publication, country, journal, indexing database, title, language of publication, type of study, number of participants, age (mean or median), type of diagnosis, outcome, clinical type, and symptoms. Microsoft^®^ Word software was used to prepare the table, and Microsoft^®^ Excel 2016 was used for organization, analysis, and symptoms exposure.

The studies' level of evidence was assessed using the Grading of Recommendations Assessment, Development and Evaluation (GRADE) system, which classifies evidence into high, moderate, low, and very low levels according to the type of study. Each type of study has its inherent level of evidence, which can be reduced or increased based on its specificities (11).

## Results

Twenty studies met the proposed inclusion criteria. The number of authors ranged from two to 34. All studies were published in 2020 and 2021 in 12 different countries. Of the included articles, 10 were in MEDLINE, 7 in Web of Science, 2 in LILACS, and 1 in SCOPUS. Most articles were published in English, followed by Spanish, and only one in Brazilian Portuguese. Regarding type of study, most were cross-sectional, case series, and cohort studies. The level of evidence ranged from moderate to very low.

The number of participants involved in the studies ranged from 14 to 341, and the main form of diagnosis was laboratory by molecular biology (RT-PCR). The main outcome of the evaluated cases was cure. Supplementary Table S1 shows the data collected from the studies included in this review (12-31).

**Figure 2:**
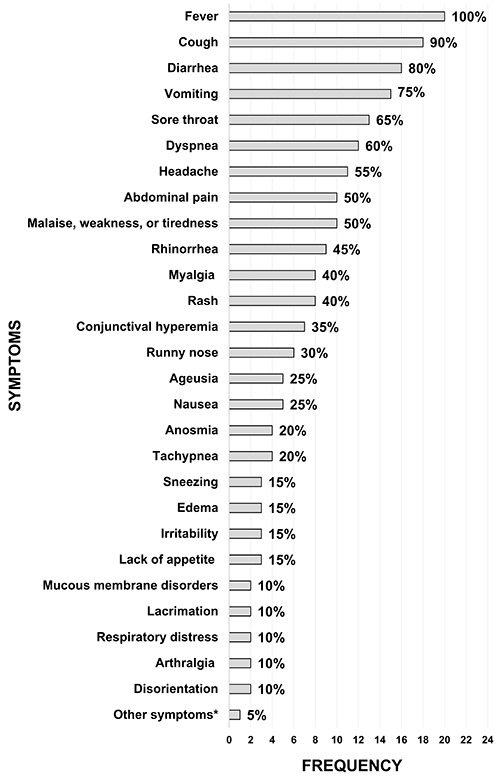
Graph showing the frequency of the onset of symptoms of COVID-19 in children in the articles included. *Symptoms that appeared in only one of the 20 studies: dysphonia, drowsiness, anorexia, dizziness, sputum, oropharyngeal hyperemia, pharyngitis, chills, odynophagia, and chest pain.

The most frequent symptom in children infected with SARS-CoV-2 was fever, which was reported in all articles. However, other symptoms such as cough, diarrhea, vomiting, sore throat, dyspnea, headache, abdominal pain, malaise, and weakness or tiredness were also reported in most studies (Figure 2).

## Discussion

China was the country that most described the symptoms of COVID-19 in children, totaling six articles included in this review (18,19,22,27,28,30). On the other hand, when considering the diversity of countries on the same continent, the American continent stands out (12-14,16,24,26,29,31). This finding highlights the magnitude of COVID-19, first detected in Asia and which, as it spread around the world, led to new studies in other regions that also investigated specificities, such as symptoms in children (1,5,12-31).

In this context, it is necessary to discuss COVID-19 in children, as the scientific literature shows that they can develop the disease at any age (32). In a systematic review that included data from 7780 children, the median age was 8.9 (± 0.5) years, with some children requiring observation or treatment in intensive care units (33).

In the present review, only 11 of the 20 studies described the clinical type of COVID-19 (13-15,19,22-24,27-30), eight of which described asymptomatic cases (13-15,22,24,27,28,30), and six described severe or critical cases (13-15,23 27,28). These findings showed that, although important, information about the clinical type and outcome is scarce in the literature and needs more attention to clarify the real impact of COVID-19 on children.

Cases requiring hospitalization were consistent with the technical note of the Brazilian Society of Pediatrics of March 17, 2021, which reported the fatality rate in children aged zero to five years hospitalized for Severe Acute Respiratory Syndrome (SRAG) related to COVID-19. This rate was 7.42% considering data collected up to epidemiological week 53 of 2020 and 5.3% considering data up to epidemiological week eight of 2021 (34). Therefore, the COVID-19 lethality was similar in the 14 studies that evaluated outcomes (12-15,20-23,26-31), in which a low number of deaths was observed, with the presence of comorbidities being a risk factor for an unfavorable prognosis.

Regarding symptoms, 37 nonspecific symptoms related to different systems were found, mainly the immune, respiratory, gastrointestinal, and neurological (13-31) systems. Fever was the most frequent manifestation of the immune system and the only one described in all studies. These data corroborate a systematic review that attempted to identify the prevalence of fever in adult (≥18 years) and pediatric (<18 years) patients with COVID-19 worldwide and found a combined prevalence of 79.43 and 45.86%, respectively (35). This indicates that fever is a symptom that occurs at different ages and should be carefully considered during screening.

The various manifestations of the respiratory system were detailed in 18 studies (12-16,18,19,21-31), with cough being the most frequent and addressed in a general way in two other studies (17,20). Dyspnea was reported in 60% of the studies, and should be taken as a warning sign for SARS. Patients may have dyspnea/respiratory distress, persistent chest pressure, reduced O_2_ saturation (less than 95% in room air), and bluish coloration of the lips or face. In children, nostril flaring, cyanosis, intercostal retraction, dehydration, and loss of appetite should also be observed in addition to the features already described (36).

Several symptoms related to the gastrointestinal system were observed (12-18,20-31), although four studies considered the manifestations without subdivision, that is, considering the entire system (15,24,26,30).

One study aimed to describe the characteristics of abdominal pain in patients with a confirmed diagnosis of COVID-19 in a pediatric hospital. Manifestations such as vomiting (73%), diarrhea (50%), and loss of appetite (20%) were described, and the location of abdominal pain, which was diffused (67%), in the umbilical region (13%), and other locations such as the right hypochondrium, right iliac fossa, hypogastrium (13%), and epigastrium (7%). It was also highlighted that diarrhea can be the first symptom of COVID-19 and that SARS-CoV-2 can be transmitted through feces (37).

The involvement of the neurological system was also reported by some studies, with headache being the main symptom (12-17,19-21,24-31). The data corroborated the findings of a study aimed to gain knowledge on the extent and severity of neurological impairment in children and adolescents associated with COVID-19 in 61 hospitals in United States (US) (38). According to the referred study, 22% of the patients had neurological impairment, indicating that this symptom is common and can lead to health complications. Thus, long-term follow-up is necessary to assess the effects of COVID-19 on child cognition and development (38).

The findings of the present review are in agreement with data from the US Centers for Disease Control and Prevention (CDC). The CDC reports that signs and symptoms of COVID-19 in children include: fatigue, headache, myalgia, cough, nasal congestion or runny nose, ageusia or anosmia, sore throat, shortness of breath or difficulty breathing, abdominal pain, diarrhea, nausea or vomiting, lack of appetite or poor diet, with the incubation period approximately the same for children and adults - from 2 to 14 days, with a mean of 6 days (39).

It has been reported, albeit less frequently, that manifestations can reach the integumentary system, causing the appearance of skin rashes and conjunctival hyperemia (13,16,17,20,21,24,26,27,29,31), and the muscular system, mainly causing myalgia (13,15,16,19,21,29-31).

Only two studies addressed MIS-C, as most were performed retrospectively, making it impossible to follow up patients after they left the health service (21,26). MIS-C is a severe condition that can involve at least two organs and systems (cardiac, renal, respiratory, hematological, gastrointestinal, dermatological, or neurological). It usually appears days or weeks after infection and is difficult to identify (40).

The present review evidenced the occurrence of symptoms that can lead to death in children, which reinforces the importance of vaccination against COVID-19 for this population (according to guidelines from health agencies) to prevent serious illness and to protect family members, including siblings who may not be eligible for vaccination. In addition, vaccination can help keep children in school and safely participate in sports, play, and other group activities (41).

In addition to vaccination, it is also necessary to carry out the diagnosis and identify the acute infection (by rapid antigen tests or molecular tests). After diagnosis, it is essential to carry out the isolation of the patient and assess the need to monitor the clinical evolution and hospitalization (42). In this review, only articles in which the laboratory diagnosis of SARS-CoV-2 was confirmed by molecular biology were included.

Among the limitations of this study is the lack of evidence on the subject because knowledge is still being produced and consolidated since it is a new subject. In addition, most included articles had a low level of evidence, because data such as sex and case scenario were not evaluated, making a broader analysis of the included cases impossible. Therefore, more studies are needed worldwide to identify more symptoms during and after COVID-19 in children and to describe their sociodemographic and clinical characteristics.

Symptoms of COVID-19 in children found in this study varied greatly and occurred uniformly in different countries. The findings of this review can contribute to the diagnosis and clinical decision-making of the healthcare team, as it provides information that facilitates the identification of COVID-19 in the target population, allowing early identification, better care, and consequently better prognosis.

Finally, this article can help improve public policies to control COVID-19 specifically targeting children, as many have been found to be asymptomatic or have only mild symptoms, acting as potential vectors of the disease in the spaces they share with adults and other children and adolescents, such as schools, the community, and the home environment.

## Supplementary Material

Click here to view [pdf].
